# Vocal Fold Leukoplakia: Which of the Classifications of White Light and Narrow Band Imaging Most Accurately Predicts Laryngeal Cancer Transformation? Proposition for a Diagnostic Algorithm

**DOI:** 10.3390/cancers13133273

**Published:** 2021-06-30

**Authors:** Wioletta Pietruszewska, Joanna Morawska, Oskar Rosiak, Agata Leduchowska, Hanna Klimza, Małgorzata Wierzbicka

**Affiliations:** 1 Department of Otolaryngology, Head and Neck Oncology, Medical University of Lodz, 22 Kopcińskiego St., 90-153 Lodz, Poland; wioletta.pietruszewska@umed.lodz.pl (W.P.); agata.leduchowska@gmail.com (A.L.); 2 Balance Disorder Unit, Department of Otolaryngology, Medical University of Lodz, 22 Kopcińskiego St., 90-153 Lodz, Poland; oskar.rosiak@umed.lodz.pl; 3 Department of Otolaryngology and Laryngological Oncology, University of Medical Sciences, 49 Stanisława Przybyszewskiego St., 60-357 Poznań, Poland; hnogala@wp.pl (H.K.); otosk2@gmail.com (M.W.)

**Keywords:** vocal fold leukoplakia, narrow band imaging, white light imaging, laryngeal cancer, low-grade dysplasia, high-grade dysplasia, leukoplakia classification, diagnostic algorithm

## Abstract

**Simple Summary:**

The management of Vocal Fold Leukoplakia, especially distinguishing malignant leukoplakia and early glottic carcinoma from benign lesions pose a challenge for laryngologists. In this two-center study we have attempted to select the most accurate classification for predicting low- and high-risk Vocal Fold Leukoplakia in White Light Imaging and Narrow Band Imaging and to establish a diagnostic algorithm with a timely referral for treatment. We have shown that both the plaque image and the microvascular pattern on the leukoplakia periphery are critical in the diagnosis of high-risk Vocal Fold Leukoplakia. The proposed algorithm combines both imaging techniques to show the added value of Narrow Band Imaging and indicates the solution for non-users.

**Abstract:**

The management of Vocal Fold Leukoplakia (VFL) remains problematic. There is no consensus on the indications or the timing for surgery. The objective was to select the most accurate classification for predicting low- and high-risk VFL in White Light Imaging (WLI) and Narrow Band Imaging (NBI) and to establish a diagnostic algorithm with a timely referral for treatment. A total of 259 VFL patients were included in the study; 186 lesions were classified as low-grade and 110 as high-grade dysplasia. The results of WLI acc. to the two-tier and the three-tier Chen 2019 classifications and NBI classifications: ELS, Ni 2011, and Ni 2019 with different cut-off points were compared with the pathological examination (HP). In WLI, the greatest agreement was obtained between type 3 of the three-tier classification and high-grade dysplasia (accuracy, specificity, and PPV: 80.4%, 92.0%, and 81.5%, respectively). Assessing VFL periphery in NBI, cut-off point 5 (Ni 2011 type V) demonstrated a higher accuracy, specificity, and PPV than 4 (83.1%, 93.6%, 85.5% and 77.4%, 74.9%, and 65.4%, respectively). In NBI, we observed higher accuracy, sensitivity, and PPV (84.1%, 93.0%, 85.2% vs. 80.7%, 81.3% and 71.3%, respectively) for cut-off point 5 (Ni 2019 type V and VI) in comparison to the cut-off point 4 group (type IV, V, and VI) (80.7%, 81.3%, 71.3%, respectively), and a higher kappa value (0.68 vs. 0.58) was obtained. We have shown that both the plaque image and the microvascular pattern on the leukoplakia periphery are critical in the diagnosis of high-risk VFL. The most accurate predictor of VFL malignant transformation in WLI is type 3 according to the Chen 2019 classification, while in NBI type V and VI according to the Ni 2019 classification.

## 1. Introduction

Approximately 90% of malignant tumors of the larynx arise from premalignant lesions, classified as leukoplakia, erythroplakia, erythroleukoplakia, and chronic laryngitis [1]. The annual incidence of the most common vocal fold leukoplakia (VFL) is estimated to be 10.2/100,000 in males and 2.1/100,000 in females [2,3,4]. The clinical term ‘leukoplakia’ refers to thick whitish or grey patches and is associated with a spectrum of histological diagnoses, from hyperplasia to malignant transformation [3,5,6,7] The potential for it to transform into laryngeal cancer (LC) is wide and estimated at 1% to 40% [4,8,9]. The most common symptom of VFL is hoarseness. An improvement of the voice quality after appropriate VFL therapy or voice preservation in asymptomatic patients is the main outcome for persons undergoing diagnosis and treatment [10]. The primary goal for the doctor is to find a balance between oncological safety and fulfilling the patients’ voice demands. Distinguishing malignant leukoplakia and early glottic carcinoma from benign lesions remains a challenge for laryngologists [11]. The following options should be considered: 1. a watch and wait policy, and observing the evolution of the lesion [12], 2. directing the patient for histopathologic examination and taking a superficial specimen or deep sampling. The HP is the ultimate determinant of the grade of dysplasia [13,14]. Superficial excision to preserve as much subepithelial tissue as possible is preferred, although deeper excision is adequate in cases with malignant potential [6].

The basis for the diagnosis of VFL is an endoscopic examination with stroboscopy as the gold standard. White Light Imaging (WLI) together with stroboscopy remain the clinical key element for detecting and assessing vocal fold lesions [6,15]. However, there are some limitations associated with conventional WLI in terms of making diagnoses [16], and although preoperative conventional video laryngoscopy remains an important examination for VFL, it is unlikely to fully agree with postoperative pathology results [12]. Approximately 50% of patients clinically diagnosed with VFL have no dysplasia found during the HP examination, but a subset of these patients will potentially undergo malignant transformation at some point in their lives [17,18]. For this reason, it has been widely discussed over the years how to best predict the pathological result based on the morphological features of leukoplakia in order to avoid both under- and overtreatment. 

To date, a number of attempts have been made to establish a scoring system for VFL and to define the predictive factors for malignant transformation. Nevertheless, VFL presents a considerable clinical challenge. Thus, for several years, biologic endoscopy techniques have attracted the attention of clinicians [19]. In recent years, biological endoscopic evaluation has made it possible to visualize neoangiogenesis, and it makes the examination and monitoring of lesions more reliable. In the past, staining with toluidine blue or Lugol’s solution was used; however, it gave a high percentage of false-positive diagnoses and required general anesthesia for direct laryngoscopy [20,21]. The use of Narrow Band Imaging (NBI) has created a new direction due to the filtering of microvessels, which indicate neoangiogenesis. This technique enabled the assessment of the pathological vessels in lesions and surrounding mucosa [22,23,24]. Compared to WLI, new information is available concerning the pathophysiology of the lesion itself, the bordering epithelium, and the immediate vicinity of the lesion [10].

Since the beginning of the 21st century, the classifications of WLI [7] and NBI imaging [22,23,24] for the assessment of VFL have been optimized to find the best possible tool for malignant transformation prediction. Validating all classifications based on one large group of patients remains an open field for research. The management of VFL remains largely controversial because there is no consensus on the indications or the timing for surgical intervention, nor for the rules of conducting the follow-up [25].

Our hypothesis assumes such an improvement in preoperative diagnostics to not expose the patient to sampling, which always carries the risk of VF damage and may result in scar formation and deterioration of voice quality. The key question is: which classification of laryngeal leukoplakia best predicts the risk of malignant transformation? Hence, an algorithm that combines two methods, i.e., WLI and NBI, should be developed to increase the value of the pre-operative evaluation and prediction. 

The main objective of this study is to select the most accurate classification for predicting the nature of low- and high-risk leukoplakia in WLI and NBI, a combination of both techniques, to show the added value of NBI and indicate the solution for non-users.

## 2. Materials and Methods

### 2.1. Patients

The study group comprised a total number of 259 patients, 212 men aged 62.08 (±9.83) years and 47 women aged 61.23 (±9.5) years with clinically diagnosed vocal fold leukoplakia who were treated at Departments of Otolaryngology, Head and Neck Oncology, Medical University of Lodz and Department of Otolaryngology and Laryngological Oncology, University of Medical Sciences, Poznań from January 2015 to March 2020. Approval for this study was granted by the Ethical Committee of Medical University of Lodz (decision no. RNN/225/19/KE, 9 April 2019)

Medical history was gathered for each patient, including age, gender, lifestyle habits (alcohol use, smoking), and comorbidities. The inclusion criteria were a diagnosis of vocal fold leukoplakia, no prior VF-related medical intervention, and procedures (surgery, radiation) and preoperative endoscopic assessment using white light endoscopy (WL) and Narrow Band Imaging (NBI). Exclusion criteria were other benign lesions (cysts, polyps, Reinke’s edema, or papilloma); a history of laryngeal surgery, trauma, or intubation; a history of radiotherapy and chemotherapy for head and neck as well as a lack of written consent from the patient.

### 2.2. Endoscopic Examination Procedure

The endoscopic examination was performed using a transnasal flexible video-endoscope with LED light (model CV-170 HD, ENF-VH, Olympus Corp, Tokyo, Japan) by four specialists (MW, WP, AL, HK). The patients were examined while in the sitting position after anesthetizing the nasal cavity. Topically applied lidocaine spray was administered to patients with a strong gag reflex. The examination started with the white light, then switched to the NBI mode. 

### 2.3. White Light Imaging (WLI)

In the WL assessment of vocal fold leukoplakia, special consideration was given to the texture (smooth, rough), thickness (flat, elevated), laterality (unilateral/bilateral), and localization (unifocal/multifocal) of the leukoplakia. Under white light mode, the lesions were categorized by means of a two-tier classification used by Ni et al. (2019) [24] and divided into: (1) low-risk leukoplakia: thin white plaque of a uniform color and a flat surface, and (2) high-risk leukoplakia: thick white plaque, a rough surface, and congestive vocal cord mucosa. 

Then, morphological assessment of the vocal fold leukoplakia was performed according to the three-tier protocol proposed by Chen et al. (2019) [7]. The morphological types of leukoplakia were categorized as: (1) flat and smooth, (2) elevated and smooth, and (3) rough. The classification describes each type in detail, taking into account the lesion’s surface, margin, and texture. Types 1 and 2 were considered low-risk lesions, and type 3 was considered high-risk.

### 2.4. Narrow Band Imaging (NBI)

#### 2.4.1. Assessment of the Immediate Surrounding of Leukoplakia Plaque According to Ni 2011 Classification

NBI endoscopy was performed in order to distinguish between malignant or benign vessel patterns. The immediate surrounding of leukoplakia plaque was assessed according to the Ni 2011 classification [22], in which types 1 and 2 were considered low-risk, whereas 4 and 5 are high-risk. Type 3 was excluded from the consideration because it is dedicated to leukoplakia itself, and the goal was to focus primarily on the assessment of the surface of the VF bordering epithelium. Types 1 and 2 were compared to 4 and 5 together (cut-off point 4) or 1 + 2 + 4 vs. 5 (cut-off point 5) to check if setting the cut-off point 5 maximizes the chance of distinguishing between low-risk and high-risk lesions.

#### 2.4.2. Assessment of the Microvascular Features of Leukoplakia According to 2015 ELS Guidelines 

The lesions were then assessed according to the protocol introduced by the European Laryngological Society (ELS), i.e., superficial vascular changes were divided into two types: (1) longitudinal and (2) perpendicular lesions. Longitudinal vessels, enlarged, static, meandering, tortuous, and dilated were classified as low-risk, while perpendicular changes of IPCLs (including enlarged vessel loops, dot-like vessels, and worm-like vessels with spiral morphology and bizarre course) represent precancerous and cancerous (high-risk) lesions [23].

#### 2.4.3. Assessment of Vocal Fold Leukoplakia According to the NBI Endoscopic Classification by Ni et al. 2019

Vocal fold leukoplakia was classified as benign (types I, II, III) or malignant (types IV, V, and VI). Then the cut-off point was moved to type V to potentially improve the accuracy of distinguishing between benign and malignant lesions.

In all patients, the surgical removal of the lesions was performed under general anesthesia. The final diagnosis was established on the basis of pathological examination of the specimens. Table 1 presents the classifications with cut-off points.

### 2.5. Histopathological Assessment

The morphology of vocal fold leukoplakia was compared with HP diagnosis for each case. Leukoplakia was divided into two types: (1) low-risk: inflammation, low-grade dysplasia; (2) high-risk: high-grade dysplasia, carcinoma in situ, and invasive carcinoma.

### 2.6. Statistical Analysis

Data were stored in a computer-based filing system and reported as absolute and relative frequencies. Statistical analysis was performed in STATISTICA 13.1 Software (Dell, USA). The cut-off values for classifications with more than two degrees were established based on the receiver operating characteristic (ROC) curve analysis, and the highest Youden’s index in each classification determined the proposed cut-off value, as per Fluss et al. [26]. To assess the diagnostic performance of the clinical classifications, the measures of occurrence (sensitivity, specificity, and accuracy) and the possibility of discriminating (positive and negative predictive values) for clinical classifications of WLI and NBI endoscopy were calculated per the determined cut-off values. To assess the level of agreement between the judgment of the WLI and NBI endoscopy, clinical classifications with more than two stages were stratified into low-risk and high-risk, using cut-off values determined by ROC curve analysis. Cohen’s kappa index (κ) was used to assess the agreement between the judgment of each lesion by clinical classifications in WLI and NBI and the histopathological results reported as low-risk and high-risk leukoplakia (WHO 2017), as previously used in similar studies [27,28]. High agreement between the ratings indicates consensus in the histopathological diagnosis and the inter-changeability of the clinical classification ratings. To interpret Cohen’s κ we followed Landis and Koch guidelines where: (κ) 0.00–0.20 indicates slight agreement, (κ) 0.21–0.40 fair agreement, (κ) 0.41–0.60 moderate agreement, (κ) 0.61–0.80 substantial agreement, and (κ) 0.81–1.00 indicates almost perfect agreement [29]. To test the null hypothesis that the probabilities of square tables comparing predictions by classifications and histopathological results are symmetrical, Bowker’s test for symmetry was used with the alpha of 0.05. If the *p*-value was less than or equal to the alpha (*p* < 0.05), the null hypothesis was rejected, and the result was considered statistically significant.

## 3. Results

A total of 259 vocal fold leukoplakia patients (212 men and 47 women) were included in the study. Bilateral vocal fold lesions were diagnosed in 37 patients (14.3%), and unilateral were diagnosed in 222 patients (85.7%). There were 170 cases of unifocal (57.4%) and 126 cases (42.6%) of multifocal lesions. In total, there were 296 specimens from 259 patients. Based on the histopathological findings, 186 lesions were classified as low-grade dysplasia and 110 lesions as high-grade dysplasia, in accordance with the WHO 2017 laryngeal dysplasia rating score [30]. Out of 296 specimens, 126 (42.57%) were multifocal leukoplastic lesions. In 41 cases, histological examination revealed invasive carcinoma with early infiltration through the basement membrane to the connective tissue stroma. For further statistical analysis, these 41 cases were included in the high-grade dysplasia group. There were former and current tobacco smokers *n* = 207 (79.9%), and 164 (63.3%) patients who admitted to occasional consumption of alcohol. 

**Figure 1:**
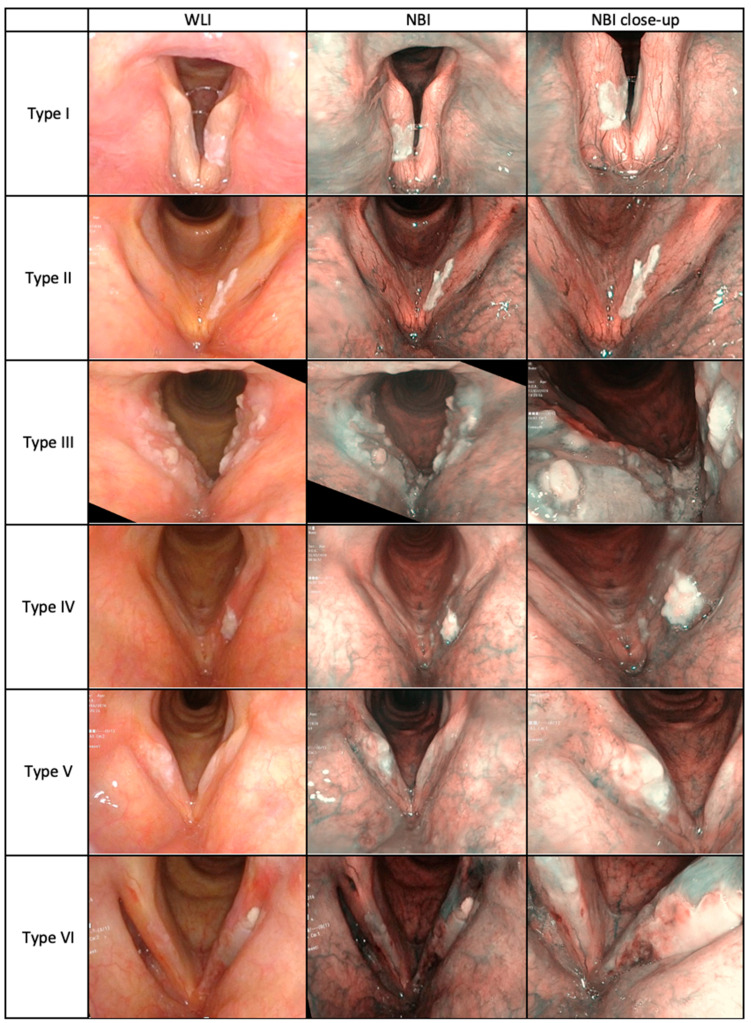
Narrow Band Imaging classification, according to Ni et al. 2019, based on the authors’ repository images from patients with vocal fold leukoplakia (*n* = 296) who were enrolled in the study.

Images of VFL in WLI and NBI according to Ni 2019 classification in the study group are presented in Figure 1. 

The results of the WL endoscopy (two-tier classification and three-tier classification according to Chen 2019) and NBI endoscopy (according to ELS classification; Ni 2011 classification; and Ni 2019 classification) were compared with the results of histopathological examination. The accuracy, sensitivity, specificity, positive and negative predictive values, ROC curve parameters: AUC and Youden index in differentiation between the histopathological results and consecutive clinical classifications are summarized in Table 2. The interrater agreement between an endoscopic examination using WLI or NBI and histopathological results is summarized in Table 3. 

Comparing the WLI classifications used, type 3 (elevated and rough leukoplakia) in the three-tier classification acc. to Chen 2019 showed the highest accuracy, specificity, and positive predictive value in the diagnosis of high-risk VFL (80.4%, 92.0%, and 81.5%, respectively) (Table 2). However, these values were lower at cut-off point 2, like those observed for the two-tier classification. These findings were confirmed by the kappa values, which were also lower for the two-tier classification, and cut-off point 2 in the three-tier classification compared to cut-off point 3 (0.44, 0.43, and 0.56, respectively). 

Analyzing the results obtained by all classification methods, WLI demonstrated lower AUC values than NBI. Determining a cut-off point for Ni 2011 merits a separate commentary. In the assessment of the leukoplakia periphery, according to Ni 2011 classification at cut-off point 4, high-grade dysplasia was detected in 85 cases (74.56%), whereas at cut-off point 5, it was confirmed in 71 patients (84.52%). Although cut-off point 5 demonstrates a similar kappa value (0.62) to cut-off point 4 (0.63), the accuracy, specificity, and PPV are significantly higher in point 5 (83.1%, 93.6%, and 85.5%, respectively) than point 4 (77.4%, 74.9%, and 65.4% respectively), indicating greater concordance between NBI and histological examination. 

Assessing leukoplakia according to Ni 2019 classification, two cut-off points were also examined, and malignancy in histological examination was confirmed in 85 out of 109 patients (77.98%) for cut-off point 4 (4 and above), and significantly more often for cut-off point 5 (in 81 out of 87 patients (93.10%)). In addition, the higher accuracy, specificity, and PPV (84.1%, 93.0%, 85.2% vs. 80.7%, 81.3%, and 71.3%, respectively) for cut-off point 5 in comparison to the cut-off point 4 group, and the higher kappa value (0.68 vs. 0.58), also emphasize its greater usefulness for predicting high-risk leukoplakia. 

When using the ELS classification in the assessment of leukoplakia periphery, we detected agreement between high-risk leukoplakia in NBI and high-grade dysplasia in histological examination, which was similar to the assessment according to Ni 2011 classification at cut-off point 4, and high-grade dysplasia was confirmed in 79 cases (72.48%) classified in NBI as perpendicular vascular changes. Comparing the ELS classification with both classifications by Ni, the ELS interrater agreement kappa value of 0.57 can be interpreted as moderate, while results obtained using both Ni classifications (Ni 2011 and Ni 2019) represent substantial agreement (0.63 and 0.68 respectively) with histological findings. 

## 4. Discussion

The authors presented and selected the most accurate classification for predicting the nature of low- and high-risk VFL out of the six WLI and NBI classifications available. We show the added value of NBI and the usefulness of the application of the latest Ni 2019 classification, which has not been researched and reported in the literature yet. 

### 4.1. WLI Classifications

WLI endoscopy remains the most common examination for the diagnosis of VFL. Three types of lesions: superficial, exophytic, and ulcerative, were distinguished [18]. The features that show the main signs of a plaque malignant transformation are the color, size, surface texture, hyperemia, thickness, edema, and symmetry of leukoplakia [31]. A combination of the four WLI variables—color, texture, size, and hyperemia—has the potential to be a comprehensive system for predicting malignancy [6,32,33]. Finally, in 2019, a new three-tier classification system was proposed [7], which seems more intuitive as it includes the assessment of the most important morphological features of VFL: surface, margin, and texture. 

### 4.2. NBI Classifications

Vascular structure alterations due to microvessel proliferation, one of the first signs of malignant transformation, can be visualized with NBI on the mucosal surface [34]. Epithelial changes such as plaque morphology (smooth, elevated, rough) and microvascular NBI characteristics play a significant role in the differential diagnosis of benign and malignant lesions [35,36,37,38,39,40]. First, a 5-degree scale of the vascularization of pathological changes (Ni 2011) was established by Ni. In order to simplify this scale, the Committee on Endoscopic Laryngeal Imaging of the European Laryngological Society (ELS) proposed a two-tier classification of the vascular patterns [23]: longitudinal vascular changes are usually benign, whereas perpendicular vascular changes are stimulated by epithelial cancerogenesis, and related to premalignant or malignant lesions [41]. This classification has recently been validated in a study by Missale et al. in a large multicenter cohort and has proved to be a highly reliable tool with good diagnostic performance in the optical biopsy setting [42]. 

The absence or limitation of mucosal wave in stroboscopy, added to NBI findings, created powerful malignancy indicators [33].

Leukoplakia plaque, due to hyperkeratotic cover, is not transparent to light. Thus, it represents a greater diagnostic problem for NBI compared to other mucosal pathologies. This was pointed out by Ni et al. in 2011 [22], who distinguished leukoplakia and classified it as a separate group, as the so-called umbrella effect hides the true pathology of the mucosa [10,39]. Later on, a six-type classification based on the morphologic characteristics of the IPCLs at the mucosal surfaces was created especially for VFL [24]. The idea behind introducing this new assessment method was to improve the accuracy of distinguishing benign and malignant leukoplakia [24]. 

In the study presented by our group, assessment of leukoplakia periphery according to the Ni 2011 classification and the European Laryngological Society classification were taken into account [22,23]. This approach was invaluable for the description of the immediate surrounding of the lesion. We confirmed the usefulness of the ELS classification in the evaluation of mucosal vascularization at the periphery of leukoplakia but found less agreement with the results of histological examination in comparison to the Ni 2011 classification using two cut-off points. 

In addition, our goal was to assess leukoplakia according to the Ni 2019 classification dedicated particularly to this premalignant laryngeal lesion [24]. High agreement between the assessment of the leukoplakia periphery according to the Ni 2011 classification and plaque plus periphery according to the Ni 2019 leukoplakia classification and HP findings was observed. However, the choice of an appropriate cut-off point is decisive. In our study, a cut-off point of 5 in the Ni 2011 classification and a cut-off point of 5 in the Ni 2019 classification confirmed the highest agreement with high-risk leukoplakia. In our study, NBI proved to be a technique of great utility. The high usefulness of assessment of the lesion itself but even more its periphery has been demonstrated. 

### 4.3. Combination of WLI and NBI Classifications

The combination of WLI and NBI might benefit in accurate diagnosis for vocal fold leukoplakia. Both NBI and Storz SPIES [43] make it possible to detect lesions smaller than 5 mm and carcinomas in situ. According to our results, the best method for predicting malignancy in leukoplakia is the three-tier classification with cut-off point 3 in WLI, and the Ni 2019 classification with cut-off point 5 in NBI. To evaluate if a synergistic effect between these two scales occurs, we searched for interaction between those classifications by means of logistic regression analysis but found none. This implies, that combining the evaluation by means of Chen 2019 and Ni 2019 classifications does not reveal new cases of high-risk VFL However, when proposing an optimal classification for clinical leukoplakia evaluation for centers without NBI, the use of the three-tier leukoplakia classification is our recommendation. The use of NBI, in turn, makes the leukoplakia assessment more reliable for predicting the risk of malignancy. This method makes it possible to extend the operation to the surrounding mucosa with pathologic vascularization visualized in NBI, but undetected in WLI. When NBI is not available and high-risk leukoplakia was diagnosed with WLI, planned sampling should be scheduled, preferably immediately, otherwise no later than 6–8 weeks. Another solution is to refer the patient to an external institution for NBI evaluation.

### 4.4. The Limitations and Strengths of the Study

WLI is an irreplaceable examination that assesses a leukoplakia plaque. It is complemented by the NBI, which evaluates the plaque, but above all, its immediate surroundings. Given that our study only demonstrated a tendency for consistency in predicting the malignancy of the lesion, we recommend that the synergism between the two scales be examined on a larger population sample.

**Figure 2:**
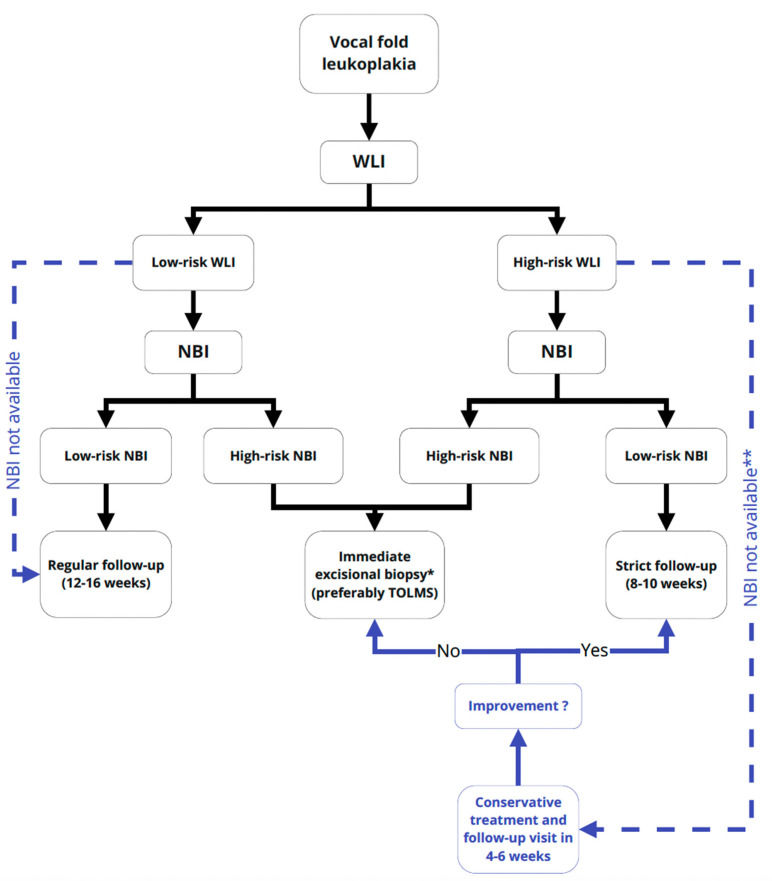
Flowchart representing the management algorithm using the three-tier classification in WLI and Ni 2019 classification in NBI in predicting high- and low-risk leukoplakia. Low-risk WLI: type 1 and 2 and high-risk WLI: type 3 acc. to Chen 2019 classification; low-risk NBI: type 1, 2, 3, 4 and high-risk WLI: type 5 and 6 acc. to Ni 2019 classification. Abbreviations used: VFL—vocal fold leukoplakia; WLI—white light imaging; NBI—Narrow Band Imaging; CT—conservative treatment; TOLMS—Transoral Laser Microsurgery; * under general anesthesia; ** referral to external NBI center.

In addition, all patients included in the study are being monitored, and long-term results will be available and reported over time. The next issue concerns the lack of NBI in some departments; thus, the proposed algorithm reflects the dual preoperative diagnostic path and cannot be narrowed down to NBI centers (Figure 2). While WLI is a standard, more canonical method, widely used and well-established, NBI is still a new tool available in few departments and has a long learning curve. 

To the best of our knowledge, so far, there have been no studies analyzing subsequent, improved classifications of VFL on one large group of patients. One large, multicenter group of VFL patients was assessed preoperatively with all available classifications and different cut-off points. 

This allowed for the selection of the two most useful methods and the definition of the cut-off point for everyday practice. To summarize, in the evaluation of leukoplakia in WLI, the greatest agreement was obtained between type 3 according to Chen 2019 classification and high-grade dysplasia. Using NBI, the cases evaluated at cut-off point 5 acc. to Ni 2019 classification of VFL were the most useful in the prognosis of probable malignancy. All of the above findings constitute a basis for the presented VLF management algorithm (Figure 2).

## 5. Conclusions

The predictor of VFL malignant transformation in WLI is type 3 according to the Chen 2019 classification, whereas in NBI, it is types V and VI, according to Ni 2019. We proved the high concordance between HP findings and assessment of the leukoplakia periphery according to Ni 2011 and, independently, plaque plus surrounding epithelium according to the Ni 2019 classifications. This indicates that it is not only the image of the plaque itself that is crucial for the diagnosis of malignant transformation but also the microvessel pattern in the leukoplakia periphery. 

## Figures and Tables

**Table 1:** Description of the classifications used with determined cut-off points (IPCLs—intraepithelial papillary capillary loops; VF—vocal fold; ELS—European Laryngological Society) [7,22,23,24].

Laryngeal Light Endoscopy	Classification	Benign Leukoplakia	Malignant Leukoplakia
White Light Endoscopy	two-tier classification	thin white plaque of a uniform color and a flat surface	thick white plaque, a rough surface, and congestive vocal fold mucosa
	three-tier classification acc. to Chen 2019	flat and smooth white plaque elevated and smooth	3. elevated and rough
Narrow Band Imaging	Ni 2011 classification: cut-off point 4	1+2 thin, oblique and arborescent interconnected vessels, IPCLs almost invisible diameter of oblique and arborescent vessels enlarged, IPCLs almost invisible	4+5 4. IPCLs recognized as small dots 5a. IPCLs appear as solid or hollow, with a brownish, speckled pattern and various shapes 5b. IPCLs appear as irregular, tortuous, line-like shapes 5c. IPCLs appear as brownish speckles or tortuous, line-like shapes with irregular distribution, scattered on the tumour surface.
	Ni 2011 classification: cut-off point 5	1+2+4	5
	ELS classification	longitudinal lesions	2. perpendicular lesions
	Ni 2019 classification: cut-off point 4	1+2+3 no IPCLs, but white plaque observed on the VF with obliquely running vessels and branching vessels indistinctly present under the white plaque white patches on VF, but neither IPCLs nor obliquely running vessels or branching vessels. IPCLs at the surface of VF mucosa where the epithelium is not covered by leukoplakia, showing small brown spots with a relatively regular arrangement and without clear boundaries. No obliquely running vessels or branching vessel can.	4+5+6 4. IPCLs on the VF, showing large brown spots and embedded at the surface of white plaque 5. IPCLs on VF, showing large brown spots, which appear at the surface of the VF mucosa outside leukoplakia with obvious boundaries. 6. IPCLs visible at the surface of the VF, characterized by large brown spots or twisted earthworm-like vessels distributed at the surface of leukoplakia and at the surface of VF epithelium outside leukoplakia.
	Ni 2019 classification: cut-off point 5	1+2+3+4	5+6

**Table 2:** Diagnostic performance of White Light Imaging (WLI) and Narrow Band Imaging (NBI) using different classifications for the detection of high-risk leukoplakia.

Endoscopic Light Imaging.	Clinical Classification	Proposed Cut-Off Point	AUC (95% CI)	Youden’s Index	Accuracy %	Sensitivity %	Specificity %	PPV %	NPV %
WLI	two-tier classification	2	0.734 (0.675; 0.793)	0.47	72.0	78.9	67.9	58.9	84.7
	three-tier classification acc. to Chen 2019	2	0.806 (0.751; 0.861)	0.46	70.9	81.7	64.7	57.4	85.8
		3	0.806 (0.751; 0.861)	0.53	80.4	60.6	92.0	81.5	80.0
NBI	ELS classification	2	0.787 (0.729; 0.844)	0.57	80.1	73.4	84.0	72.7	84.4
	Ni 2011 classification	4	0.856 (0.809; 0.902)	0.56	77.4	81.7	74.9	65.4	87.5
		5	0.856 (0.809; 0.902)	0.59	83.1	65.1	93.6	85.5	82.2
	Ni 2019 classification	4	0.869 (0.826; 0.912)	0.61	80.7	79.8	81.3	71.3	87.4
		5	0.869 (0.826; 0.912)	0.62	84.1	68.8	93.0	85.2	83.7

**Table 3:** Summary of interrater agreement between endoscopic results using White Light Imaging (WLI) or Narrow Band Imaging (NBI) and histopathological examination results using the proposed cut-off points/values according to the clinical classification used in the study.

Laryngeal Endoscopy	Clinical Classification	Cut-Off Point	Low-Risk VFL Cases	High-Risk VFL Cases	Cohens Kappa (κ) Coefficient Value	Bowker’s Test for Symmetry (*p* Value)
White Light Imaging	two-tier classification	2	150	146	0.44	<0.001
	three-tier classification acc. to Chen 2019	2	141	155	0.43	<0.001
		3	215	81	0.56	<0.001
Narrow Band Imaging	ELS classification	2	186	110	0.57	0.896
	Ni 2011 classification	4	181	115	0.63	<0.001
		5	211	85	0.62	0.404
	Ni 2019 classification	4	176	120	0.58	0.152
		5	208	88	0.68	<0.001
Total VFL cases in HP			187	109		

## Abbreviations

NBI	narrow band imaging
WLI	white light imaging
VF	vocal fold
VFL	vocal fold leukoplakia
HP	histopathology
ELS	European Laryngological Society
IPCLs	Intraepithelial papillary capillary loops
CT	conservative treatment
TOLMS	Transoral Laser Microsurgery

## References

[1] Gale N., Michaels L., Luzar B., Poljak M., Zidar N., Fischinger J., Cardesa A. (2009). Current review on squamous intraepithelial lesions of the larynx. Histopathology.

[2] Peng J., Li H., Chen J., Wu X., Jiang T., Chen X. (2018). Differences in gene expression profile between vocal cord Leukoplakia and normal larynx mucosa by gene chip. J. Otolaryngol. Head Neck Surg..

[3] Ahn A., Wang L., Slaughter J.C., Nguyen A.M., Ossoff R.H., Francis D.O. (2016). Serial full-thickness excision of dysplastic vocal fold leukoplakia: Diagnostic or therapeutic?. Laryngoscope.

[4] Bouquot J.E., Gnepp D.R. (1991). Laryngeal precancer: A review of the literature, commentary, and comparison with oral leukoplakia. Head Neck.

[5] Li C., Zhang N., Wang S., Cheng L., Wu H., Chen J., Chen M., Shi F. (2018). A new classification of vocal fold leukoplakia by morphological appearance guiding the treatment. Acta Otolaryngol..

[6] Fang T.J., Lin W.N., Lee L.Y., Young C.K., Lee L.A., Chang K.P., Liao C.T., Li H.Y., Yen T.C. (2016). Classification of vocal fold leukoplakia by clinical scoring. Head Neck.

[7] Chen M., Li C., Yang Y., Cheng L., Wu H. (2019). A morphological classification for vocal fold leukoplakia. Braz. J. Otorhinolaryngol..

[8] Ma L.-J., Wang J., Xiao Y., Ye J.-Y., Xu W., Yang Q.-W. (2013). Clinical classification and treatment of leukokeratosis of the vocal cords. Chin. Med. J. (Engl)..

[9] Isenberg J.S., Crozier D.L., Dailey S.H. (2008). Institutional and comprehensive review of laryngeal leukoplakia. Ann. Otol. Rhinol. Laryngol..

[10] Klimza H., Jackowska J., Tokarski M., Piersiala K., Wierzbicka M. (2017). Narrow-band imaging (NBI) for improving the assessment of vocal fold leukoplakia and overcoming the umbrella effect. PLoS ONE.

[11] Lu G., Guo W., Zhang Q., Song X. (2021). Endoscopic diagnosis value of narrow band imaging Ni classification in vocal fold leukoplakia and early glottic cancer. Am. J. Otolaryngol..

[12] Ni X.G., Wang G.Q., Hu F.Y., Xu X.M., Xu L., Liu X.Q., Chen X.S., Liu L., Ren X.L., Yang Y. (2019). Clinical utility and effectiveness of a training programme in the application of a new classification of narrow-band imaging for vocal cord leukoplakia: A multicentre study. Clin. Otolaryngol..

[13] Lee Y.C., Eun Y.-G., Park I.-S. (2018). The Value of I-Scan Image-Enhanced Endoscopy in the Diagnosis of Vocal Cord Leukoplakia. J. Korean Soc. Laryngol. Phoniatr. Logop..

[14] Cui W., Xu W., Yang Q., Hu R. (2017). Clinicopathological parameters associated with histological background and recurrence after surgical intervention of vocal cord leukoplakia. Medicine (United States).

[15] Mannelli G., Cecconi L., Gallo O. (2016). Laryngeal preneoplastic lesions and cancer: Challenging diagnosis. Qualitative literature review and meta-analysis. Crit. Rev. Oncol. Hematol..

[16] Sun C., Han X., Li X., Zhang Y., Du X. (2017). Diagnostic Performance of Narrow Band Imaging for Laryngeal Cancer: A Systematic Review and Meta-analysis. Otolaryngol. Head Neck Surg..

[17] Bartlett R.S., Heckman W.W., Isenberg J., Thibeault S.L., Dailey S.H. (2012). Genetic characterization of vocal fold lesions: Leukoplakia and Carcinoma. Laryngoscope.

[18] Lee D.H., Yoon T.M., Lee J.K., Lim S.C. (2015). Predictive factors of recurrence and malignant transformation in vocal cord leukoplakia. Eur. Arch. Oto-Rhino-Laryngol..

[19] Shoffel-Havakuk H., Lahav Y., Meidan B., Haimovich Y., Warman M., Hain M., Hamzany Y., Brodsky A., Landau-Zemer T., Halperin D. (2017). Does narrow band imaging improve preoperative detection of glottic malignancy? A matched comparison study. Laryngoscope.

[20] Olofsson J. (1950). TOLUIDINE blue. Whats. New.

[21] Mishra A., Nilakantan A., Sahai K., Sethi A., Singh S., Datta R. (2012). Contact Endoscopy - A promising tool for evaluation of laryngeal mucosal lesions. J. Laryngol. Voice.

[22] Ni X.G., He S., Xu Z.G., Gao L., Lu N., Yuan Z., Lai S.Q., Zhang Y.M., Yi J.L., Wang X.L. (2011). Endoscopic diagnosis of laryngeal cancer and precancerous lesions by narrow band imaging. J. Laryngol. Otol..

[23] Arens C., Piazza C., Andrea M., Dikkers F.G., Tjon Pian Gi R.E.A., Voigt-Zimmermann S., Peretti G. (2016). Proposal for a descriptive guideline of vascular changes in lesions of the vocal folds by the committee on endoscopic laryngeal imaging of the European Laryngological Society. Eur. Arch. Oto-Rhino-Laryngol..

[24] Ni X.G., Zhu J.Q., Zhang Q.Q., Zhang B.G., Wang G.Q. (2019). Diagnosis of vocal cord leukoplakia: The role of a novel narrow band imaging endoscopic classification. Laryngoscope.

[25] Kim C.M., Chhetri D.K. (2020). Triological Best Practice: When Is Surgical Intervention Indicated for Vocal Fold Leukoplakia?. Laryngoscope.

[26] Fluss R., Faraggi D., Reiser B. (2005). Estimation of the Youden Index and its associated cutoff point. Biom. J..

[27] Piazza C., Cocco D., Del Bon F., Mangili S., Nicolai P., Majorana A., Bolzoni Villaret A., Peretti G. (2010). Narrow band imaging and high definition television in evaluation of oral and oropharyngeal squamous cell cancer: A prospective study. Oral Oncol..

[28] Warrens M.J. (2015). Five Ways to Look at Cohen’s Kappa. J. Psychol. Psychother..

[29] Landis J.R., Koch G.G. (1977). Landis amd Koch1977_agreement of categorical data. Biometrics.

[30] El-Naggar A.K. (2017). Squamous cell carcinoma. WHO Classification of Head and Neck Tumors.

[31] Campo F., Ralli M., Di Stadio A., Greco A., Pellini R., de Vincentiis M. (2020). Role of Narrow Band Imaging Endoscopy in Preoperative Evaluation of Laryngeal Leukoplakia: A Review of the Literature. Ear Nose Throat J..

[32] Young C.K., Lin W.N., Lee L.Y., Lee L.A., Hsin L.J., Liao C.T., Li H.Y., Chen I.H., Fang T.J. (2015). Laryngoscopic characteristics in vocal leukoplakia: Inter-rater reliability and correlation with histology grading. Laryngoscope.

[33] Rzepakowska A., Sobol M., Sielska-Badurek E., Niemczyk K., Osuch-Wójcikiewicz E. (2020). Morphology, Vibratory Function, and Vascular Pattern for Predicting Malignancy in Vocal Fold Leukoplakia. J. Voice.

[34] Irjala H., Matar N., Remacle M., Georges L. (2011). Pharyngo-laryngeal examination with the narrow band imaging technology: Early experience. Eur. Arch. Oto-Rhino-Laryngol..

[35] Šifrer R., Rijken J.A., Leemans C.R., Eerenstein S.E.J., van Weert S., Hendrickx J.J., Bloemena E., Heuveling D.A., Rinkel R.N.P.M. (2018). Evaluation of vascular features of vocal cords proposed by the European Laryngological Society. Eur. Arch. Oto-Rhino-Laryngol..

[36] Ansari U.H., Wong E., Smith M., Singh N., Palme C.E., Smith M.C., Riffat F. (2019). Validity of narrow band imaging in the detection of oral and oropharyngeal malignant lesions: A systematic review and meta-analysis. Head Neck.

[37] Weller M.D., Nankivell P.C., McConkey C., Paleri V., Mehanna H.M. (2010). The risk and interval to malignancy of patients with laryngeal dysplasia; a systematic review of case series and meta-analysis. Clin. Otolaryngol..

[38] Staníková L., Šatanková J., Kučová H., Walderová R., Zeleník K., Komínek P. (2017). The role of narrow-band imaging (NBI) endoscopy in optical biopsy of vocal cord leukoplakia. Eur. Arch. Oto-Rhino-Laryngol..

[39] Huang F., Yu J., Zhang F., He C., Li S., Shao J. (2017). The usefulness of narrow-band imaging for the diagnosis and treatment of vocal fold leukoplakia. Acta Otolaryngol..

[40] Popek B., Bojanowska-Poźniak K., Tomasik B., Fendler W., Jeruzal-Świątecka J., Pietruszewska W. (2019). Clinical experience of narrow band imaging (NBI) usage in diagnosis of laryngeal lesions. Otolaryngol. Pol..

[41] Mehlum C.S., Rosenberg T., Dyrvig A.K., Groentved A.M., Kjaergaard T., Godballe C. (2018). Can the Ni classification of vessels predict neoplasia? A systematic review and meta-analysis. Laryngoscope.

[42] Missale F., Taboni S., Carobbio A.L.C., Mazzola F., Berretti G., Iandelli A., Fragale M., Mora F., Paderno A., Del Bon F. (2021). Validation of the European Laryngological Society classification of glottic vascular changes as seen by narrow band imaging in the optical biopsy setting. Eur. Arch. Oto-Rhino-Laryngol..

[43] Abdullah B., Rasid N.S.A., Lazim N.M., Volgger V., Betz C.S., Mohammad Z.W., Hassan N.F.H.N. (2020). Ni endoscopic classification for Storz Professional Image Enhancement System (SPIES) endoscopy in the detection of upper aerodigestive tract (UADT) tumours. Sci. Rep..

